# Characterization of lysine crotonylation-related lncRNAs for prognostic assessment and immune response in glioma

**DOI:** 10.3389/fphar.2025.1573694

**Published:** 2025-06-30

**Authors:** Miaomiao Song, Juan Xu, Zhonghao Gui, Yun Wu, Feifei Wang, Hongmei Sheng, Xueyong Huang, Junyu Qian, Haotian Qin, Ying Wang

**Affiliations:** ^1^ Department of Encephalopathy, Second Affiliated Hospital of Anhui University of Chinese Medicine, Hefei, China; ^2^ Department of Oncology, Chaohu Hospital of Anhui Medical University, Hefei, China; ^3^ Department of Neurology, Lu’an Hospital of Anhui Medical University, Lu’an, China; ^4^ National & Local Joint Engineering Research Center of Orthopaedic Biomaterials, Peking University Shenzhen Hospital, Shenzhen, China; ^5^ Famous TCM Studio of Ying WANG, Anhui University of Chinese Medicine, Hefei, China

**Keywords:** glioma, LCRlncRNA, immune microenvironment, chemoresistance, biomarkers

## Abstract

**Background:**

Glioma is a highly aggressive brain tumor with limited therapeutic options and poor prognosis. While immune checkpoint inhibitors and molecular therapies have emerged, effective biomarkers for patient stratification remain scarce. Long non-coding RNAs (lncRNAs) associated with lysine crotonylation (LCRlncRNAs) have been implicated in cancer progression, but their role in glioma remains largely unexplored.

**Methods:**

Transcriptomic and clinical data from The Cancer Genome Atlas (TCGA) glioma cohort were analyzed to identify prognostic LCRlncRNAs. A multigene risk score model was constructed using univariate Cox, LASSO, and multivariate Cox regression analyses. Functional enrichment analyses (GO, KEGG, GSEA) and immune landscape profiling (CIBERSORT, ssGSEA, ESTIMATE) were performed to explore potential mechanisms. Associations with immune checkpoint expression, tumor mutational burden (TMB), and microsatellite instability (MSI) were also assessed. In addition, RT-qPCR, EdU, Transwell, and xenograft experiments, as well as qPCR, Western blot, serum ELISA, and immunohistochemistry (IHC) analyses, were conducted to validate the functional and mechanistic roles of the representative LCRlncRNA POLR2J4.

**Results:**

Six LCRlncRNAs were identified as independent prognostic factors, and the risk score model stratified patients into high- and low-risk groups with distinct survival outcomes. The high-risk group exhibited enriched immunosuppressive features, including increased regulatory T cells, M2 macrophages, and elevated expression of immune checkpoints (e.g., PD-L1, CTLA4). TIDE analysis indicated poor immunotherapy response in high-risk patients. Drug sensitivity analysis revealed that high-risk patients were more sensitive to DNA-damaging agents such as cisplatin. Functional assays confirmed that POLR2J4 promotes glioma proliferation, migration, and cisplatin resistance. Mechanistically, POLR2J4 knockdown reduced the expression of drug resistance genes (ABCB1, ABCC1, BCL2), decreased serum levels of IL-6 and TGF-β1, and downregulated TGF-β1 and PD-L1 in tumor tissues, highlighting its role in establishing an immunosuppressive, drug-resistant microenvironment.

**Conclusion:**

Our study demonstrates that LCRlncRNAs are closely linked to glioma prognosis, immune microenvironment remodeling, and therapeutic response. The LCRlncRNA-based risk model provides a promising tool for prognostic evaluation and personalized therapy design in glioma.

## Introduction

Glioma is the most prevalent type of primary intracranial tumor, accounting for approximately 30% of all brain neoplasms. Based on malignancy, gliomas are classified into low-grade gliomas (LGG) and glioblastomas (GBM), the latter being highly aggressive and associated with a median survival of less than 15 months despite maximal surgical resection and chemoradiotherapy (Ostrom et al., 2023; Dymova et al., 2021; Rong et al., 2022). The poor prognosis of GBM is largely attributed to its extensive inter- and intra-tumoral heterogeneity, acquired resistance to temozolomide (TMZ), and an immunosuppressive tumor microenvironment (Goenka et al., 2021; Tan et al., 2020). While targeted therapies and immune checkpoint inhibitors such as anti–PD-1/PD-L1 antibodies have shown promise in certain glioma subsets, their clinical efficacy remains inconsistent, underscoring the urgent need for novel prognostic biomarkers and therapeutic targets.

Long non-coding RNAs (lncRNAs), defined as transcripts exceeding 200 nucleotides without protein-coding potential, are increasingly recognized as critical regulators of glioma biology. They participate in chromatin remodeling, transcriptional control, and post-transcriptional gene regulation, thereby influencing tumor proliferation, invasion, immune evasion, and drug resistance (Ahmad et al., 2024; Zhu et al., 2022). Recent studies have revealed that lncRNAs can modulate treatment response by affecting the tumor immune landscape and key signaling pathways such as PI3K/Akt and NF-κB (Yang et al., 2023). However, the epigenetic mechanisms linking lncRNAs to immune modulation and chemoresistance in glioma remain poorly understood.

Lysine crotonylation (Kcr), a recently discovered post-translational modification, adds a crotonyl group to lysine residues and has emerged as a key epigenetic mark associated with active transcription, metabolic regulation, and immune response (Zhang et al., 2022; Westerveld et al., 2025; Yang S. et al., 2024). Dysregulated crotonylation has been implicated in tumor progression by altering oncogenic signaling and immune cell activity. In glioma, components of the crotonylation machinery—such as crotonyltransferases and de-crotonylases (e.g., SIRT3)—have been associated with tumorigenic pathways and immune checkpoint expression (Yuan et al., 2023). Despite growing interest, the interaction between crotonylation-related regulatory mechanisms and lncRNAs in glioma remains largely unexplored.

In this study, we aimed to investigate lysine crotonylation–related lncRNAs (LCRlncRNAs) and their roles in glioma prognosis, immune modulation, and chemotherapeutic response. By integrating transcriptomic and clinical data from TCGA, we identified six LCRlncRNAs with significant prognostic value and established a robust risk score model. Notably, one representative lncRNA—POLR2J4—was selected for in-depth experimental validation. We demonstrated that POLR2J4 promotes glioma cell proliferation, migration, and resistance to cisplatin both *in vitro* and *in vivo*. Moreover, immune cell infiltration analysis and drug sensitivity prediction revealed that high-risk patients exhibit a distinct immunosuppressive phenotype and enhanced responsiveness to DNA-damaging agents. Our findings highlight the clinical significance of LCRlncRNAs as novel biomarkers and potential therapeutic targets in glioma, and provide new insights into the epigenetic–immune axis underlying treatment resistance, as confirmed by qPCR, ELISA, and IHC analyses.

## Materials and methods

### Data sources and preprocessing

Gene expression datasets and comprehensive clinical data for glioma were obtained from The Cancer Genome Atlas (TCGA) database (https://portal.gdc.cancer.gov/) (Tomczak et al., 2015). The dataset included gene expression profiles for 429 glioma patients and 3 normal tissue samples. The corresponding clinical-pathological information is provided in Supplementary Table 1 and includes details such as Age, Gender, Grade, IDH status, 1p/19q codeletion, MGMT promoter status, and overall survival (OS) time and status. All data used in this study were standardized to Transcripts Per Million (TPM) units, and their distribution was confirmed to be approximately normal. Data visualization and exploration were carried out using R software (v4.4.1) with the “ggplot2” package, and gene expression data matrices were constructed for further analysis. A Wilcoxon test was applied for differential expression analysis. Additionally, 18 lysine crotonylation-related genes (LCRGs) identified in previous studies were used for further analysis, as outlined in Supplementary Table 2 (Jiang et al., 2023).

### Lysine crotonylation-related lncRNA detection

After extracting mRNA and lncRNA expression data from the TCGA database, Pearson correlation coefficients were used to analyze the relationships between the expression levels of lncRNAs and the identified LCRGs. Correlation thresholds were set at >0.55 and p < 0.001. The “limma” R package was used for identifying significant associations between LCRGs and lncRNAs, and the co-expression data were visualized using “ggplot2” and “ggalluvial” tools for better understanding of the relationships between the lncRNAs and LCRGs.

### Construction and Validation of the prognostic model based on LCRlncRNAs

To identify LCRlncRNAs associated with patient survival, univariate Cox regression was performed with a significance threshold of p < 0.05, and the results were visualized using forest plots. Further refinement of the selected LCRlncRNAs was conducted through LASSO-Cox regression analysis, using 10-fold cross-validation and a significance threshold of p < 0.05. This process was repeated 1,000 times to prevent overfitting. The expression of LCRGs and lncRNAs was used to select 6 LCRlncRNAs significantly correlated with patient survival. The results were visualized using the “ggplot2” package. R packages used in this analysis included “survival,” “caret,” “glmnet,” “survminer,” and “timeROC.” Based on the multivariate Cox regression analysis, a risk score for each patient was calculated using the following formula: Riskscore = ∑_i_ Cofficient (LCRlncRNAs_i_) × Expression (LCRlncRNAs_i_). The dataset was randomly divided into a training cohort and a testing cohort in a 1:1 ratio, with corresponding survival data for each patient. Glioma patients from TCGA were categorized into low-risk and high-risk subtypes based on the average risk score. Kaplan-Meier survival analysis was then performed to compare the overall survival rates between these two subtypes, followed by time-dependent ROC analysis to assess the accuracy of the predictive model. Additionally, clinical tissue samples (n = 24) were used as an independent validation set to evaluate the accuracy of the prognostic risk model. These analyses were conducted using R packages such as “survival,” “survminer,” and “pheatmap.”

### Prognostic nomogram construction and validation

Univariate and multivariate Cox regression analyses were performed to identify prognostic factors, with the results displayed using forest plots generated by the “forestplot” R package, which includes hazard ratios (HRs), 95% confidence intervals (CIs), and p-values for each variable. To further evaluate the predictive performance of the LCRlncRNA-based risk score, the model’s performance was compared with clinical features (e.g., Age, Gender, Grade, IDH status, 1p/19q codeletion, MGMT promoter status). The receiver operating characteristic (ROC) curves for 1-year, 3-year, and 5-year survival were generated, along with time-dependent AUC curves and the concordance index (C-index). These clinical variables were also used in subgroup survival analysis to assess their influence on prognosis. Based on the results from the multivariate Cox proportional hazards analysis, a nomogram was developed to predict 1-year, 3-year, and 5-year overall survival (OS) for glioma patients. The nomogram was then validated by plotting calibration curves for each time point to evaluate the consistency between predicted and observed outcomes. Additionally, ROC curves for 1-year, 3-year, and 5-year survival were constructed to assess the model’s predictive accuracy. The R packages utilized in these analyses included “survival,” “survminer,” “timeROC,” “dplyr,” “rms,” “regplot,” “survcomp,” and “pec.”

### Functional enrichment analysis

Differentially expressed genes (DEGs) between high- and low-risk groups were identified using the “limma” package in R. The analysis was conducted on adjusted p-values to correct for false positive results, with a significance threshold of “Adjusted P < 0.05” and a fold change threshold of “|log2FC| ≥ 1” to define differentially expressed genes. To explore the functional roles of the DEGs, Gene Ontology (GO) enrichment analysis and Kyoto Encyclopedia of Genes and Genomes (KEGG) pathway enrichment analysis were performed using the “clusterProfiler” R package (Yu et al., 2012). GO analysis categorized the DEGs into three main categories: Biological Process (BP), Cellular Component (CC), and Molecular Function (MF). KEGG analysis provided insights into the biological pathways that were enriched in the high- and low-risk groups. Additionally, Gene Set Enrichment Analysis (GSEA) was performed using the GSEA tool (http://software.broadinstitute.org/gsea/index.jsp) (Powers et al., 2018) to identify potential biological pathways associated with the high- and low-risk groups.

### Immune cell infiltration analysis

To investigate the relationship between LCRlncRNAs and immune cell infiltration, the ssGSEA (single-sample Gene Set Enrichment Analysis) and CIBERSORT algorithms were employed to compute immune cell infiltration levels. These analyses were visualized using the “ggplot2” R package. The immune cell infiltration levels between the high-risk and low-risk groups were compared using the Wilcoxon test. For immune scoring, the “immunedeconv” R package and the CIBERSORT algorithm (Newman et al., 2015) were used to evaluate the degree of immune cell infiltration in both high- and low-risk groups. In addition, ssGSEA (Hänzelmann et al., 2013) implemented in the “GSVA” R package (version 1.46.0) was used to quantify the infiltration levels of various immune cell types, as well as the accumulation of 24 common immune cell types. The Wilcoxon rank-sum test was applied to assess the differences in immune cell infiltration between the two groups. Finally, the “estimate” R package (version 1.0.13) was used to calculate immune cell abundance (immune score), stromal cell infiltration (stromal score), and the overall ESTIMATE score for each patient.

### Immunotherapy response analysis

To evaluate the potential for immunotherapy response in glioma patients, a correlation analysis was performed between the risk score and various immune checkpoint-related genes. The expression levels of immune checkpoint genes between high and low-risk groups were compared, as well as the relationship between prognostic LCRlncRNAs and immune checkpoint genes. Genes of interest in this study included CD274 (PD-L1), CTLA4, HAVCR2, LAG3, PDCD1, PDCD1LG2, TIGIT, and SIGLEC15. These immune checkpoint-related genes were visualized using the “ggplot2” and “pheatmap” R packages. Further, the prognostic significance of the combination of the risk score with three key immune checkpoints (CD274, CTLA4, PDCD1) was analyzed to predict patient outcomes. The Tumor Immune Dysfunction and Exclusion (TIDE) algorithm was employed to predict the potential immune checkpoint-blocking response, and the results were visualized using the “ggplot2” package in R. The TIDE data distribution was also compared between high and low-risk groups to assess the potential for immune checkpoint blockade therapy in glioma patients.

### Biomarker prediction and potential drug screening for immunotherapy

The tumor mutation burden (TMB) and microsatellite instability (MSI) were analyzed between high and low-risk groups in the TCGA cohort using the Wilcoxon rank-sum test. Additionally, the correlation between the risk score and human leukocyte antigen (HLA) genes, as well as DNA mismatch repair (MMR) genes, was evaluated using Spearman’s method. The “survminer” R package’s “surv_cutpoint” function was used to calculate the optimal TMB and MSI cutoff values, which were then used to divide glioma patients into high and low TMB and MSI subgroups. Prognostic analysis was conducted via Kaplan-Meier survival curves to assess the impact of these features on overall survival (OS). Subsequently, the OS of patients in the high-risk and low-risk groups was compared across the four subgroups (high TMB, low TMB, high MSI, and low MSI) using Kaplan-Meier survival analysis. Additionally, drug sensitivity was assessed between the high-risk and low-risk groups using the “limma,” “ggpubr,” and “pRRophetic” R packages (Geeleher et al., 2014). This analysis aimed to identify potential drugs for glioma treatment and compare the sensitivity of high-risk and low-risk groups to these drugs.

### Human sample collection

All tissue samples for this study were provided by Chaohu Hospital of Anhui Medical University, and included 24 pairs of glioma tissues and adjacent normal tissues, along with follow-up data for each patient. The tissue samples were embedded in 10% formalin for preservation. Pathological examination was performed on all samples to confirm the diagnosis of glioma. This study was approved by the Ethics Committee of Chaohu Hospital of Anhui Medical University (Approval No. KYXM202410008), and all patients provided informed consent. The experiments were conducted in accordance with the relevant guidelines and regulations.

### Cell culture and transfection

Normal human astrocytes (NHA) and glioblastoma cell lines (U87, U251, and T98G) were obtained from the American Type Culture Collection (ATCC, Manassas, VA, United States). Cells were cultured in Dulbecco’s Modified Eagle Medium (DMEM) with the following formulations: NHA in DMEM (Gibco, Cat# 11965–092), and glioblastoma cells in DMEM (Gibco, Cat# 11995–065). All media were supplemented with 10% fetal bovine serum (FBS; Gibco, Cat# 10099–141) and 1% penicillin-streptomycin (PS; Gibco, Cat# 15140–122). U87 and U251 cells were seeded in six-well plates and grown to 60%–70% confluency. To silence POLR2J4, a long non-coding RNA (lncRNA), cells were transfected with short hairpin RNAs (shRNAs) targeting POLR2J4 (GeneRulor, Zhuhai, China) using Lipofectamine 3,000 (Invitrogen, United States), following the manufacturer’s protocol. Total RNA was extracted 48 h after transfection for RT-qPCR to assess knockdown efficiency. All experiments were performed in triplicate.

### RNA extraction and RT-qPCR

Total RNA was extracted using the Quick-RNA MiniPrep Kit (Zymo Research, R1054). Target gene expression was detected using the miScript SYBR Green PCR Kit (Qiagen, Germany) on a LightCycler 96 real-time PCR system (Roche Diagnostics GmbH, Mannheim, Germany). Relative expression levels were quantified using the 2-^△△CT^ method, with GAPDH as the reference gene.

### EdU staining

Cells were seeded in 24-well plates and incubated with 10 μM EdU for 30 min at 37°C using the Click-iT™ EdU Alexa Fluor™ 488 Imaging Kit (Invitrogen, United States). Following incubation, cells were fixed with 4% paraformaldehyde for 20 min and permeabilized with 0.5% Triton X-100 for 10 min at room temperature. Nuclei were counterstained with DAPI (1 μg/mL) for 5 min. Fluorescent images were captured using a Leica TCS SP8 confocal microscope, and EdU-positive cells were quantified using ImageJ.

### Transwell migration and invasion assays

Cell migration and invasion assays were performed using 24-well Transwell chambers with 8-μm pore size inserts (Corning, Cat# 3422) coated with or without Matrigel (Corning, Cat# 354480). For each assay, 5 × 10^4^ cells suspended in serum-free DMEM were added to the upper chamber, while medium containing 10% FBS was placed in the lower chamber as a chemoattractant. After 24 h of incubation at 37°C, non-migrated cells on the upper surface were gently removed. The cells that migrated or invaded to the lower surface were fixed with methanol, stained with 0.1% crystal violet, and counted under a microscope at ×100 magnification in five randomly selected fields.

### Xenograft mouse model

A total of 1 × 10^6^ U87 cells stably transduced with either sh-POLR2J4 or control shRNA (sh-N.C.) were suspended in 100 μL of serum-free DMEM and subcutaneously injected into the flanks of 4-week-old male BALB/c nude mice (n = 3 per group). Tumor growth was monitored every 3 days, and tumor volume was calculated using the formula V = (length × width^2^)/2. After 21 days, all mice were euthanized, and tumors were harvested, photographed, and weighed for analysis. All animal procedures were approved by the Institutional Animal Care and Use Committee (IACUC) of Shenzhen Peking University–The Hong Kong University of Science and Technology Medical Center, and conducted in accordance with institutional and national ethical guidelines.

### Serum ELISA analysis

Blood samples were collected from tumor-bearing nude mice by cardiac puncture at the endpoint of the experiment. Serum was separated by centrifugation at 3,000 rpm for 10 min and stored at −80°C until analysis. The concentrations of interleukin-6 (IL-6) and transforming growth factor-β1 (TGF-β1) in the serum were measured using commercially available mouse ELISA kits (Multi Sciences Biotech, Hangzhou, China; catalog nos. EK206/3 and EK183/3, respectively) according to the manufacturer’s instructions. Briefly, serum samples and standards were added to 96-well plates pre-coated with target-specific antibodies, followed by incubation with horseradish peroxidase–conjugated detection antibodies. After adding the TMB substrate solution, the colorimetric reaction was stopped with 2M sulfuric acid, and absorbance was measured at 450 nm using a microplate reader (Bio-Rad). Cytokine concentrations were calculated based on standard curves generated using known concentrations of recombinant proteins.

### Immunohistochemistry (IHC) analysis

Tumor tissues were harvested from xenograft-bearing mice, fixed in 10% neutral-buffered formalin, embedded in paraffin, and sectioned at 4 μm thickness. For antigen retrieval, tissue sections were heated in citrate buffer (pH 6.0) using a microwave oven for 15 min. Endogenous peroxidase activity was blocked by incubation with 3% hydrogen peroxide for 10 min. Sections were then blocked with 5% bovine serum albumin (BSA) for 30 min at room temperature. Primary antibodies against TGF-β1 (1:200, Abcam, ab215715) and PD-L1 (1:100, Cell Signaling Technology, #13684) were applied overnight at 4°C. After washing, sections were incubated with HRP-conjugated secondary antibodies (Servicebio, China) for 30 min at room temperature. Immunoreactivity was visualized using a DAB substrate kit (Servicebio, China) and counterstained with hematoxylin. Images were captured using an Olympus BX51 microscope, and semi-quantitative analysis of immunostaining intensity was performed using ImageJ software by calculating the integrated optical density (IOD) per unit area (IOD/mm^2^).

### Statistical analysis

All statistical analyses were conducted using R software (https://www.r-project.org/). Each section of the study was analyzed with specific datasets, R packages, and databases. A p-value of less than 0.05 was considered statistically significant (*p < 0.05, **p < 0.01, ***p < 0.001). The statistical methodologies employed included survival analysis, correlation analysis, and comparison tests to assess the significance of the observed relationships and predictions.

## Results

### Identification of prognostic LCRlncRNAs and construction of prognostic features

**FIGURE 1:**
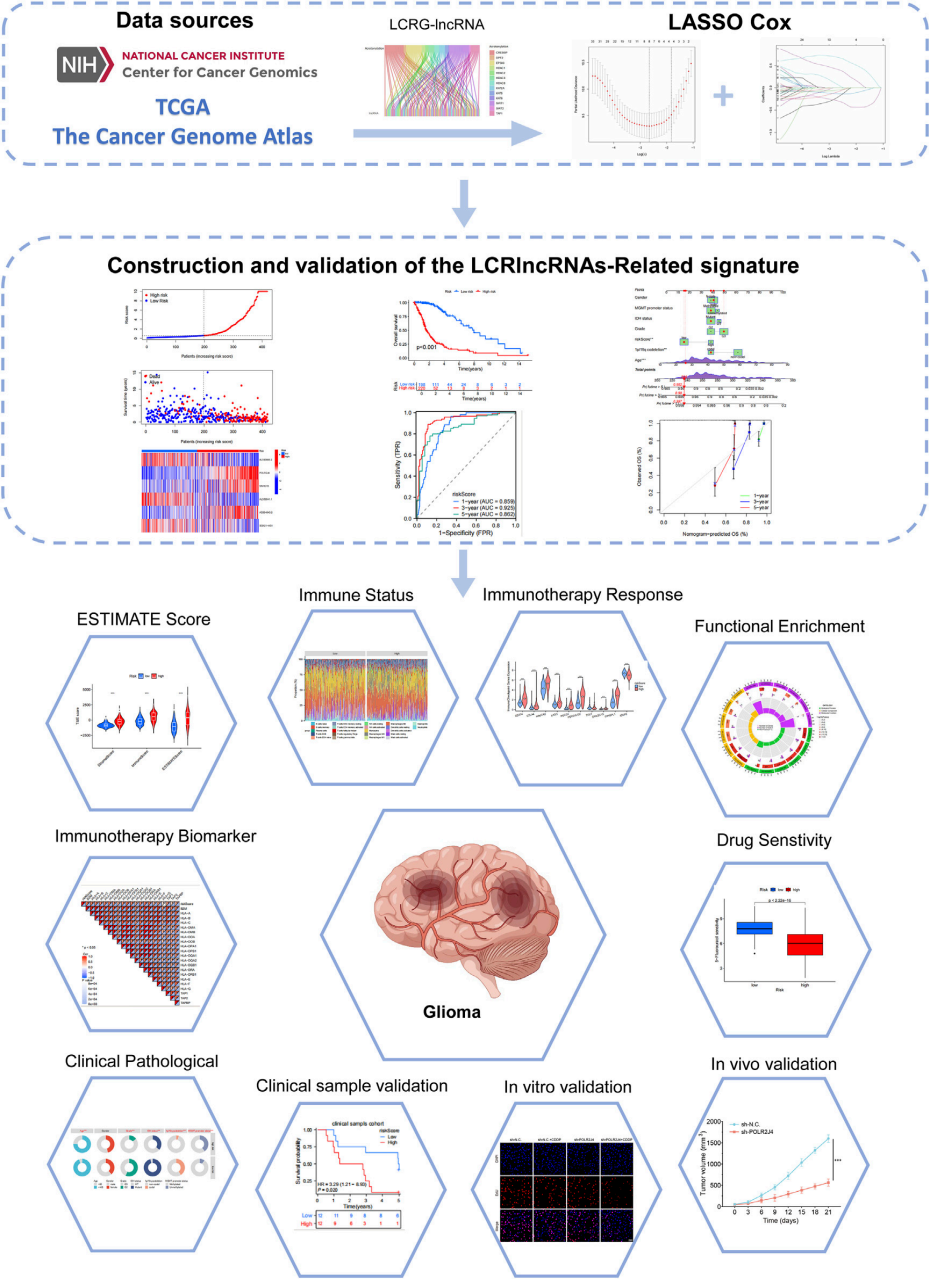
Flowchart of the present study.

**FIGURE 2:**
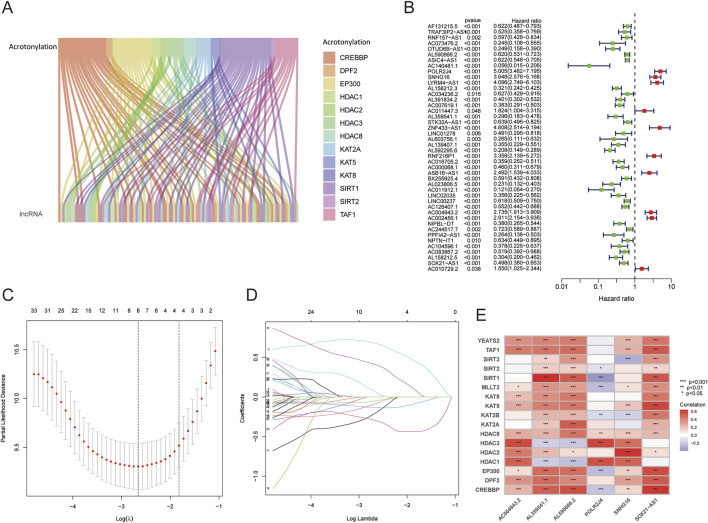
Identification of Prognostic-Related LCRlncRNAs and Construction of Prognostic Features. **(A)** Co-expression analysis of lysine crotonylation-related genes and lncRNAs. **(B)** Prognostic value of DRlncRNAs based on univariate Cox regression analysis (P < 0.05). **(C)** LASSO coefficient plot for prognostic LCRlncRNAs. **(D)** Cross-validation error rate plot (10-fold). **(E)** Correlation heatmap between LCRlncRNAs and LCRGs.

The flowchart of this study is shown in Figure 1. We analyzed the expression levels of 18 LCRGs from the TCGA-glioma dataset, which included 429 glioma patient samples and 3 normal tissue samples. Co-expression analysis was performed to identify LCRG-related lncRNAs (Figure 2A), resulting in 115 lncRNAs significantly associated with lysine crotonylation (Supplementary Table 3). Six LCRG-lncRNAs significantly correlated with the survival prognosis of glioma patients (P < 0.05) were identified through univariate Cox regression (Figure 2B). A prognostic model was then constructed using LASSO Cox regression based on these LCRG-lncRNAs (Figures 2C,D). Multivariate Cox regression analysis was conducted, and the correlation between the six identified LCRG-lncRNAs was visualized in a heatmap (Figure 2E). Risk scores were determined based on the expression levels of these six LCRlncRNAs. The OS (Overall Survival) analysis for glioma patients yielded the following risk score formula:

RiskScore=AL590666.2 ∗(−0.239)+POLR2J4 ∗ 0.919+SNHG16 ∗ 0.635+AL359541.1∗(−0.716)+AC004943.2 ∗ 0.531+SOX21−AS1∗(−0.304).

### Expression and prognostic value of LCRlncRNAs

To validate the expression and diagnostic utility of prognostic LCRlncRNAs in glioma, we analyzed TCGA transcriptome data. Compared to normal tissues, the expression levels of AL590666.2, POLR2J4, SNHG16, AL359541.1, AC004943.2, and SOX21-AS1 were significantly upregulated in glioma tissues (Supplementary Figure 1A). ROC curve analysis demonstrated that all these lncRNAs exhibited high diagnostic accuracy, with AUC values greater than 0.8 (Supplementary Figure 1B). Kaplan-Meier survival analysis showed that high expression of POLR2J4, SNHG16, and AC004943.2 was significantly associated with poorer survival outcomes in terms of PFI (Progression-Free Interval) and DSS (Disease-Specific Survival) (P < 0.001) (Supplementary Figure 1C,D). Conversely, low expression of AL590666.2, AL359541.1, and SOX21-AS1 was associated with worse outcomes (P < 0.001) (Supplementary Figure 1C,D). Therefore, these LCRlncRNAs exhibit significant expression abnormalities in glioma tissues and are closely associated with patient prognosis, making them potential biomarkers and therapeutic targets for early glioma diagnosis and treatment.

### LCRlncRNAs-based risk model demonstrates robust prognostic power in glioma

**FIGURE 3:**
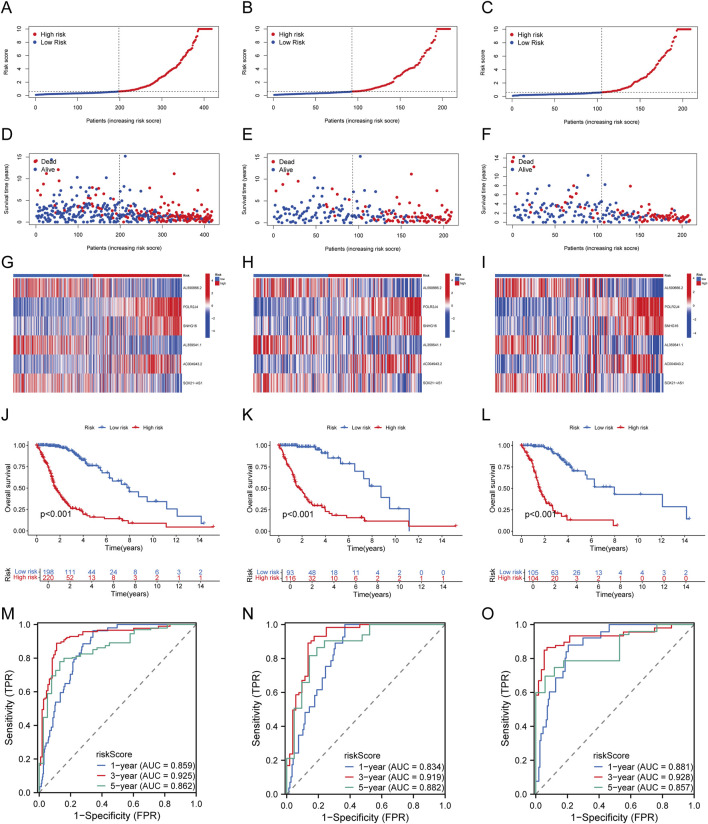
Establishment and Validation of Prognostic Features for LCRlncRNAs. **(A–C)** Distribution of risk scores for each patient in the TCGA-glioma cohort, training cohort, and testing cohort. **(D–F)** Distribution of overall survival status for each patient in the TCGA-glioma cohort, training cohort, and testing cohort. **(G–I)** Heatmap showing the expression of six prognostic LCRlncRNAs in the TCGA-glioma cohort, training cohort, and testing cohort. **(J–L)** Kaplan-Meier survival curves for high- and low-risk groups in the TCGA-glioma cohort, training cohort, and testing cohort. **(M–O)** Time-dependent ROC curves for 1-, 3-, and 5-year OS in the TCGA-glioma cohort, training cohort, and testing cohort.

The 418 TCGA-glioma samples were randomly divided into a training set and a validation set. Patients were grouped into high-risk and low-risk groups based on the median risk score in all, training, and testing sets. As the risk score increased, patients’ mortality risk and the likelihood of shorter survival time increased (Figures 3A–F). The heatmap displayed the distinct expression patterns of lncRNAs between the high-risk and low-risk groups (Figures 3G–I). Kaplan-Meier survival curves revealed that glioma patients with high-risk scores had significantly lower overall survival rates than those with low-risk scores in all, training, and testing groups (P < 0.05) (Figures 3J–L). The ROC curves for predicting OS at 1, 3, and 5 years in all groups had AUC values of 0.859, 0.925, and 0.862, respectively (Figure 3M). Similarly, in the training set, the AUC values were 0.834, 0.919, and 0.882 at 1, 3, and 5 years, respectively (Figure 3N). In the testing set, the AUC values were 0.881, 0.928, and 0.857 at 1, 3, and 5 years, respectively (Figure 3O). These findings confirm the efficacy of our risk score model, which can effectively predict glioma OS. Thus, the LCRlncRNAs prognostic features hold strong potential for clinical application in glioma prognosis.

### Analysis of the association between clinical pathological features and risk score

We categorized the patients from the TCGA-glioma cohort based on clinical features such as age, gender, grade, IDH status, 1p/19q co-deletion, and MGMT promoter status. As shown in Supplementary Figure 2A and Supplementary Table 4, we analyzed the association between the risk score and these clinicopathological characteristics. The risk score was significantly correlated with age, grade, IDH status, 1p/19q co-deletion, and MGMT promoter status. Subgroup survival analysis indicated that the high-risk group had a significantly shorter overall survival time across all categories (age, grade, IDH status, 1p/19q co-deletion, and MGMT promoter status) (Supplementary Figure 2B). These findings suggest that these factors play a crucial role in determining survival outcomes for glioma patients and should be considered in treatment strategy development.

### Development and validation of a prognostic nomogram

**FIGURE 4:**
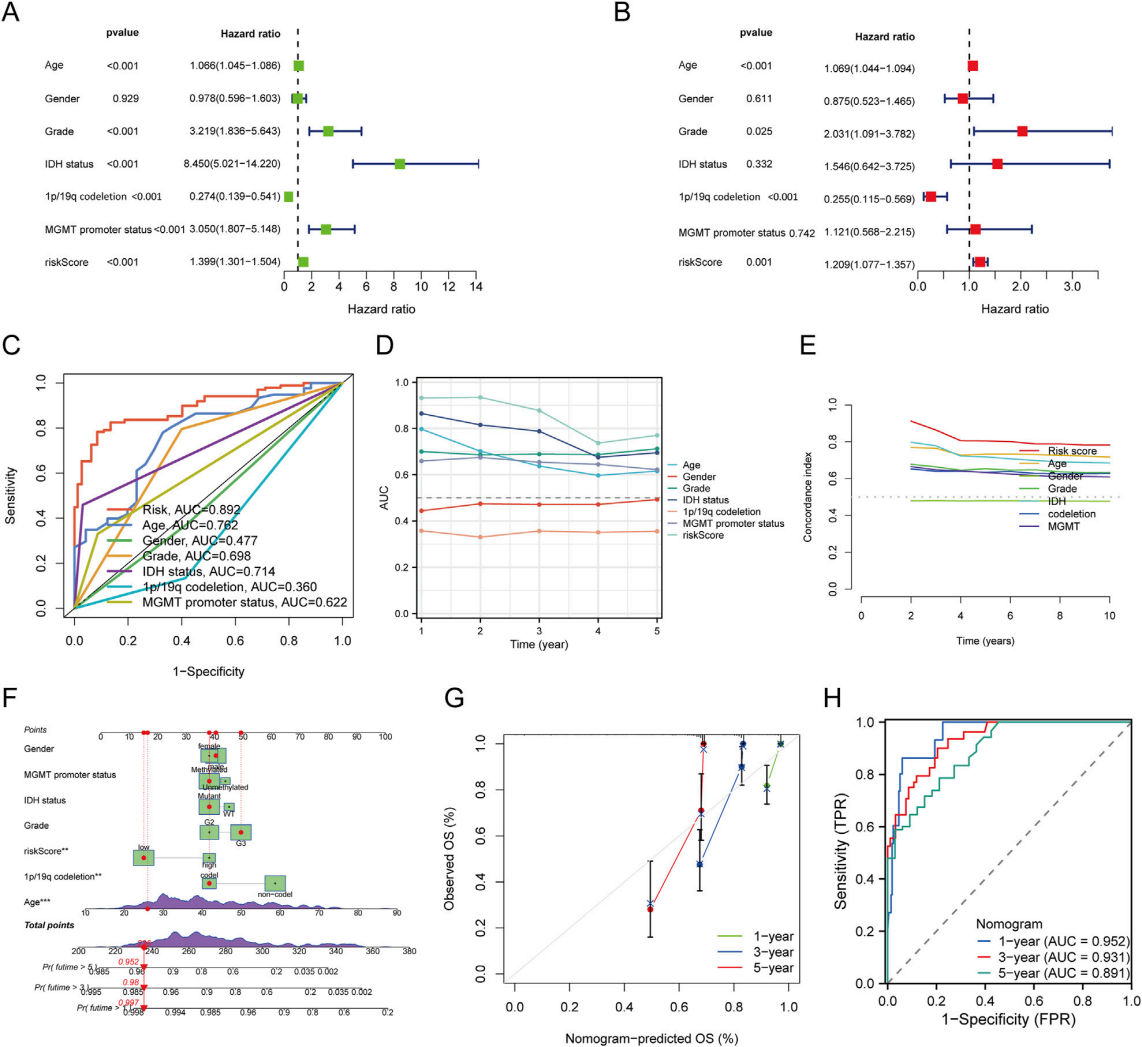
Construction and Validation of a Predictive Nomogram. **(A,B)** Univariate and multivariate Cox regression analyses for clinical variables in glioma. **(C)** ROC curves for risk score and different clinical parameters (Age, Gender, Grade, IDH status, 1p/19q codeletion, MGMT promoter status). **(D)** Time-dependent AUC curve showing the OS prediction performance of the risk score. **(E)** Concordance index values for the risk score and other clinical variables. **(F)** Nomogram predicting 1-, 3-, and 5-year OS in glioma patients. **(G)** Calibration curve for the OS nomogram model in the discovery group (diagonal dotted line represents the ideal nomogram). **(H)** ROC curves for predicting 1-, 3-, and 5-year OS.

We first performed both univariate and multivariate Cox regression analyses to construct a nomogram incorporating the risk score and other prognostic clinical factors. In the univariate Cox regression analysis, age (p < 0.001, HR = 1.066 [1.045–1.086]), gender (p < 0.001, HR = 3.219 [1.836–5.643]), IDH status (p < 0.001, HR = 8.450 [5.021–14.220]), 1p/19q co-deletion (p < 0.001, HR = 0.274 [0.139–0.541]), MGMT promoter status (p < 0.001, HR = 3.050 [1.807–5.148]), and risk score (p < 0.001, HR = 1.399 [1.301–1.504]) were found to be significantly associated with the overall survival (OS) of glioma patients. In the multivariate Cox regression analysis, age (p < 0.001, HR = 1.069 [1.044–1.094]), gender (p = 0.025, HR = 2.031 [1.091–3.782]), 1p/19q co-deletion (p < 0.001, HR = 0.255 [0.115–0.569]), and risk score (p = 0.001, HR = 1.209 [1.077–1.357]) were identified as independent prognostic factors for OS(Figures 4A,B). The ROC curve analysis showed that the risk score model had the highest AUC (0.892) for predicting OS (Figure 4C). We also plotted time-dependent AUC curves for all indicators in the TCGA cohort, demonstrating their predictive performance for OS (Figure 4D). Furthermore, the concordance index (C-index) for the risk score was significantly higher than that of other clinical factors (Figure 4E). We then integrated the risk score with the clinical factors to develop a nomogram for predicting the 1-, 3-, and 5-year OS of glioma patients (Figure 4F). The calibration curve of the nomogram showed good consistency between predicted and actual observed values (Figure 4G). The ROC AUC values for 1-, 3-, and 5-year predictions were 0.952, 0.931, and 0.891, respectively (Figure 4H). These results highlight the clinical value of the nomogram in predicting survival rates.

### Functional enrichment analysis of high and low-risk LCRlncRNAs

**FIGURE 5:**
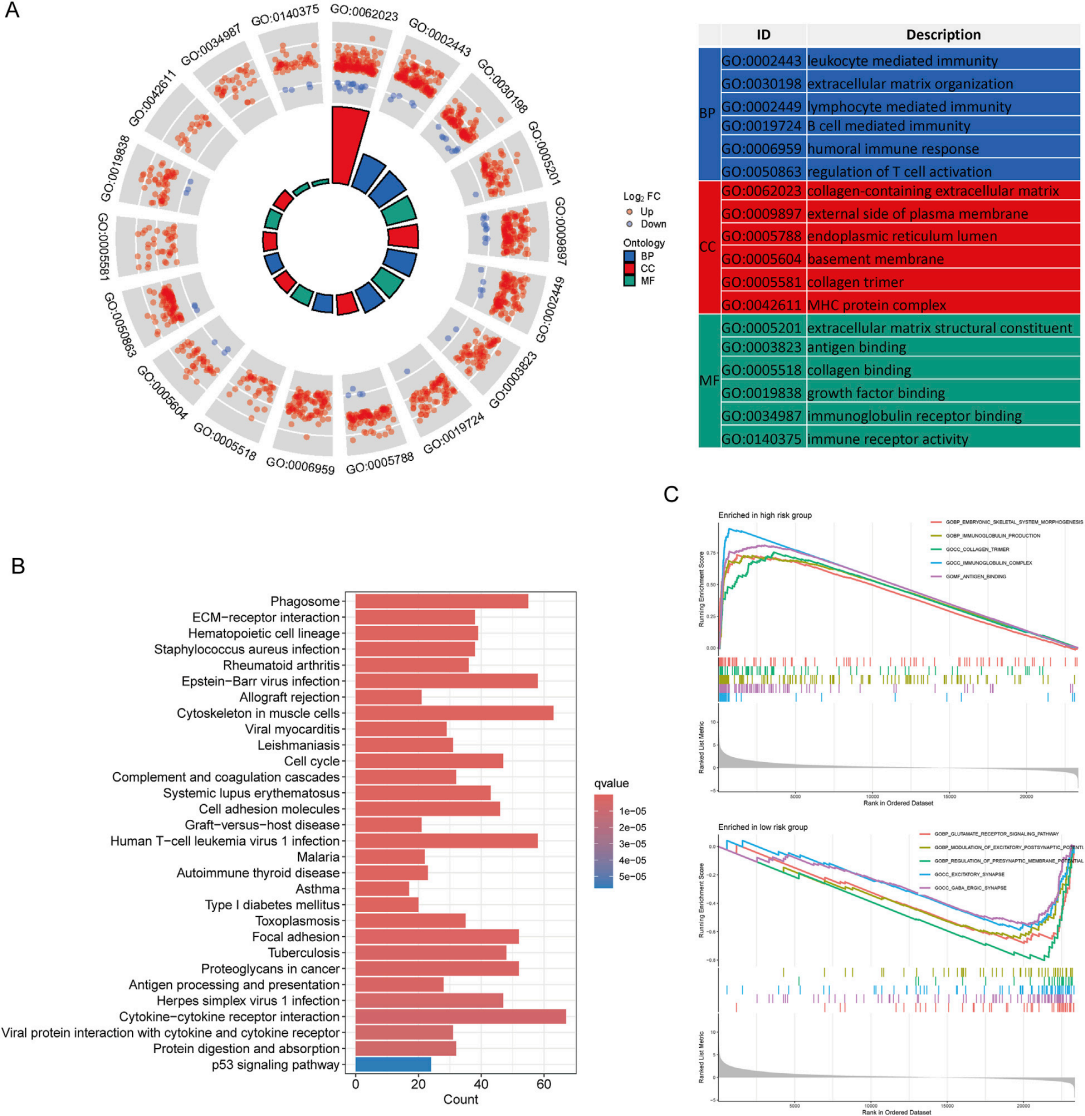
Functional Enrichment Analysis Between Low- and High-Risk Groups. **(A)** GO enrichment results. **(B)** KEGG pathways. **(C)** GSEA analysis.

Following the selection criteria, we identified 3,042 DEGs between high-risk and low-risk LCRlncRNA groups. GO analysis revealed that these DEGs were predominantly enriched in BP, including leukocyte-mediated immunity, extracellular matrix organization, lymphocyte-mediated immunity, B cell-mediated immunity, humoral immune response, and regulation of T cell activation. CC were mainly enriched in the collagen-containing extracellular matrix, external side of plasma membrane, endoplasmic reticulum lumen, basement membrane, collagen trimer, and MHC protein complex. MF included extracellular matrix structural constituent, antigen binding, collagen binding, growth factor binding, immunoglobulin receptor binding, and immune receptor activity (Figure 5A). KEGG pathway analysis showed significant enrichment in pathways such as the cell cycle, TNF signaling, NF-kappa B signaling, PI3K-Akt signaling, IL-17 signaling, NOD-like receptor signaling, Toll-like receptor signaling, Fanconi anemia pathway, and chemokine signaling (Figure 5B). Additionally, GSEA revealed that genes in the high-risk group were mainly associated with embryonic skeletal system morphogenesis, immunoglobulin production, collagen trimer formation, immunoglobulin complex formation, and antigen binding. In contrast, genes in the low-risk group were primarily involved in glutamate receptor signaling, modulation of excitatory postsynaptic potential, regulation of presynaptic membrane potential, excitatory synapses, and GABAergic synapses (Figure 5C).

### Immune cell infiltration analysis

**FIGURE 6:**
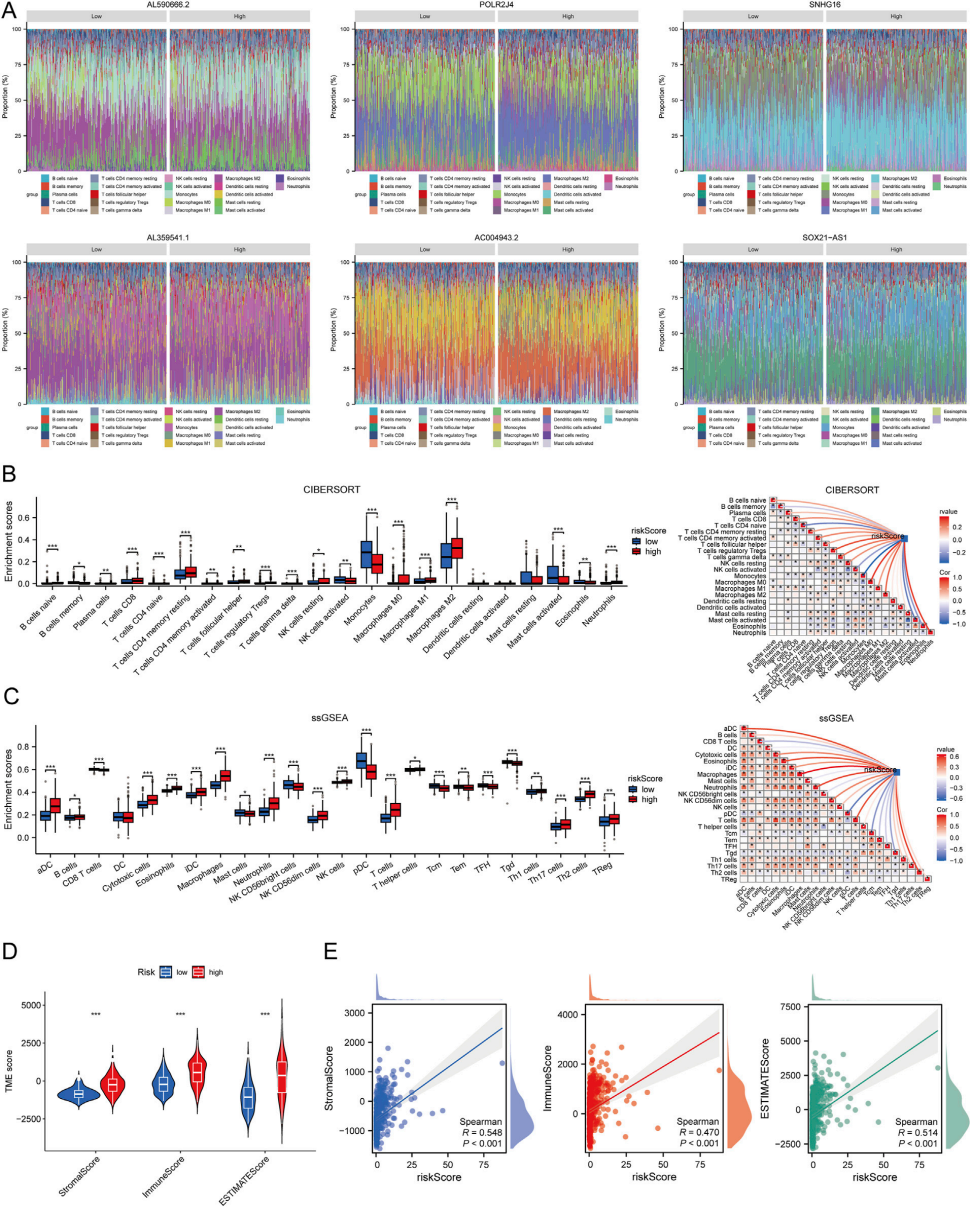
Relationship Between Risk Score and Immune Infiltration in the Tumor Microenvironment. **(A)** Percentage abundance of tumor-infiltrating immune cells between high and low expression groups of the six prognostic LCRlncRNAs; different colors represent different immune cell types, with the x-axis representing samples and the y-axis representing the immune cell content percentage in each sample. **(B)** Correlation analysis between risk score and immune infiltration based on the CIBERSORT algorithm. **(C)** Correlation analysis between risk score and immune infiltration based on the ssGSEA algorithm. **(D)** Differences in ESTIMATE scores between high and low-risk score groups. **(E)** Correlation analysis between risk score and ESTIMATE.

We performed a correlation analysis between the risk score and immune cell infiltration using different algorithms: CIBERSORT, ssGSEA, and ESTIMATE. The stacked bar chart (Figure 6A) illustrates the distribution of immune infiltration scores between high and low expression groups of six prognostic LCRlncRNAs. The CIBERSORT algorithm revealed that memory B cells, monocytes, and eosinophils were significantly more expressed in the low-risk group, while CD4 memory resting T cells, regulatory T cells (Tregs), follicular helper T cells, gamma-delta T cells, resting NK cells, and M2 macrophages showed higher expression in the high-risk group. Correlation analysis further demonstrated that the risk score was positively correlated with Tregs and M2 macrophages, but negatively correlated with monocytes, eosinophils, and memory B cells (Figure 6B). The ssGSEA method indicated that mast cells, NK CD56bright cells, pDC, central memory T cells (Tcm), effector memory T cells (Tem), follicular helper T cells (TFH), and gamma-delta T cells (Tgd) were more highly expressed in the low-risk group, whereas macrophages and Tregs were more prevalent in the high-risk group. The risk score showed positive correlations with macrophages and Tregs and negative correlations with pDC, Tcm, Tgd, CD8 T cells, Tem, and TFH (Figure Figure6C). Additionally, we analyzed the relationship between the risk score and the three ESTIMATE scores. The results indicated significantly higher ImmuneScore, StromalScore, and ESTIMATEscore in the high-risk group compared to the low-risk group (P < 0.001) (Figure 6D). This suggests that the high-risk group is characterized by a more active immune microenvironment (Figure 6E).

### Immune therapy response analysis

The immune checkpoint gene expression differences between high-risk and low-risk groups were analyzed based on eight genes selected for their established or emerging relevance to glioma immunotherapy. These genes—CD274 (PD-L1), CTLA4, PDCD1 (PD-1), PDCD1LG2 (PD-L2), HAVCR2 (TIM-3), LAG3, SIGLEC15, and ITPRIPL1—represent both classical and next-generation immunotherapeutic targets. CD274, PDCD1, and CTLA4 are widely used clinical markers, with evidence showing that PD-L1–positive patients benefit from PD-1 and CTLA4 co-inhibition. LAG3 and HAVCR2 are linked to immunotherapy resistance, while SIGLEC15 and ITPRIPL1 are novel targets associated with TAM polarization and T cell suppression. PDCD1LG2 (PD-L2) has been implicated in immune escape via a compensatory mechanism with PD-L1, supporting the rationale for dual checkpoint blockade. Expression analysis revealed that all eight genes were significantly upregulated in the high-risk group (Supplementary Figure 3A). Correlation analysis showed a positive association between the risk score and expression levels of these checkpoints (Supplementary Figure 3B). Furthermore, patients with high expression of these immune checkpoint genes had significantly worse survival outcomes (Supplementary Figure 3C). To evaluate the potential response to immune checkpoint blockade (ICB), TIDE analysis indicated that the high-risk group exhibited higher TIDE scores, suggesting reduced responsiveness to ICB therapy (Supplementary Figure 3D). Additionally, the high-risk group had increased abundance of MDSCs, CAFs, and M2 TAMs (Supplementary Figure 3E–G), reinforcing its immunosuppressive phenotype. These findings suggest that high-risk patients may be more likely to exhibit immune evasion and poor response to ICB, highlighting the clinical importance of incorporating risk scores into immunotherapy decision-making.

### Immune therapy biomarker prediction

The high-risk group exhibited significantly higher TMB and MSI scores compared to the low-risk group (Supplementary Figure 4A). Prognostic analysis revealed that patients with higher TMB and MSI scores had a poorer survival outcome than those with lower TMB and MSI scores (Supplementary Figure 4B). Furthermore, we conducted a survival analysis combining the risk score with TMB and MSI. Patients were divided into four subgroups, and survival assessment indicated that the overall survival (OS) was significantly worse in the high TMB/MSI + high risk score group compared to the low TMB/MSI + low risk score group (P < 0.001) (Supplementary Figure 4C). Additionally, we evaluated the correlation between the risk score and human leukocyte antigen (HLA) and mismatch repair (MMR) genes. The results showed a significant positive correlation between the risk score and both HLA and MMR genes (Supplementary Figure 4D).

### Drug sensitivity analysis

Drug sensitivity analysis revealed that the high-risk group exhibited significantly lower IC50 values for a wide range of chemotherapy drugs compared to the low-risk group. Drugs with notably lower IC50 values in the high-risk group included 5-Fluorouracil, Alpelisib, Bortezomib, Cisplatin, Gemcitabine, Irinotecan, and many others (Supplementary Figure 5). These results suggest that patients with high-risk scores may be more sensitive to these chemotherapeutic agents, which could potentially inform treatment strategies for glioma patients.

### Validation via cell experiments and clinical samples

**FIGURE 7:**
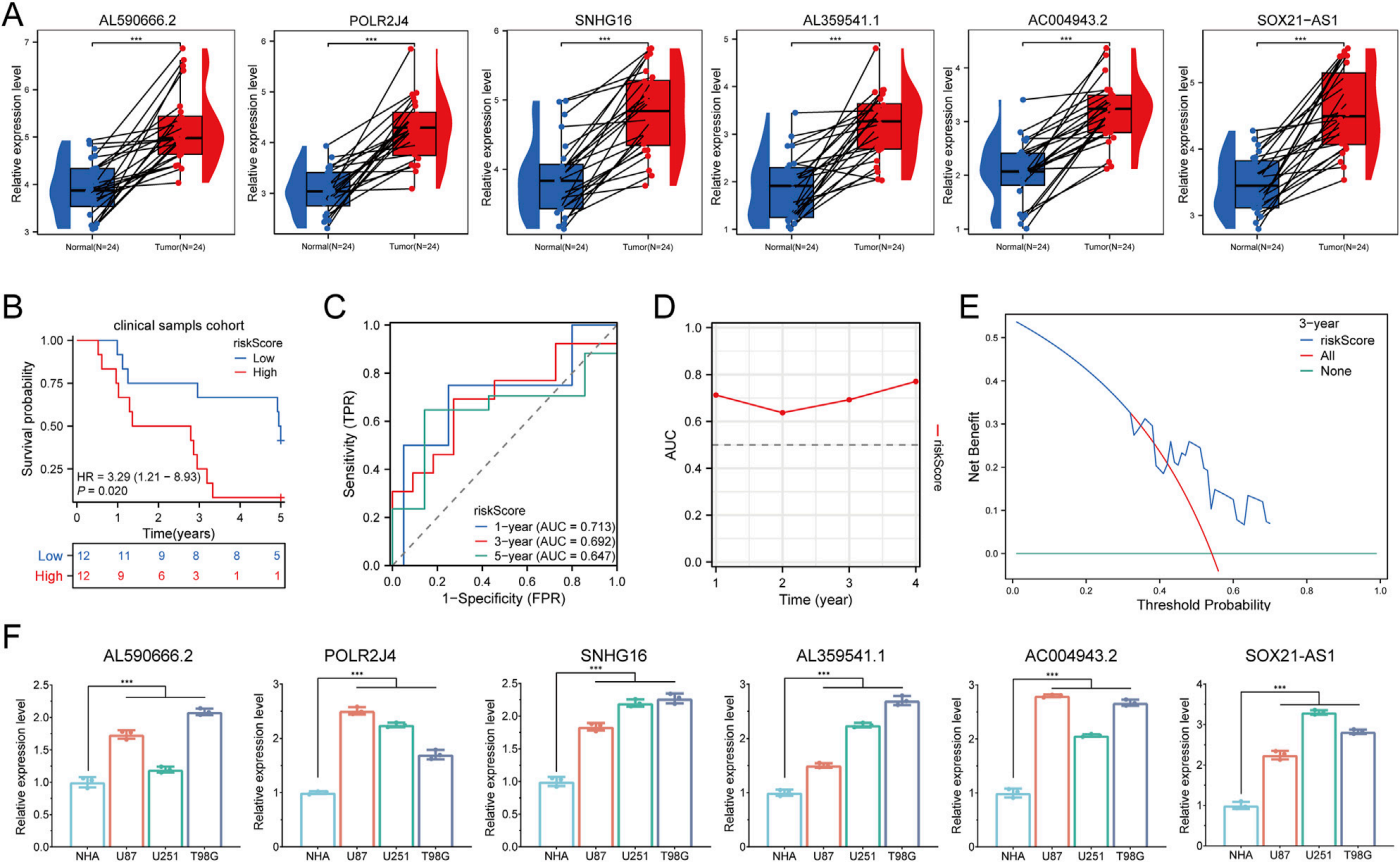
Clinical Sample and Cell Experiment Validation. **(A)** Relative expression of LCRlncRNAs in normal tissues and glioma tissues. **(B)** Overall survival curve for high- and low-risk glioma patients. **(C)** Time-dependent ROC curves for LCRlncRNAs at 1, 3, and 5 years. **(D)** Time-dependent AUC curves. **(E)** Decision curve analysis for 3-year OS in clinical samples. **(F)** Differential expression of LCRlncRNAs in glioma cell lines and corresponding normal cell lines.

We utilized RT-qPCR to assess the expression of six prognostic LCRlncRNAs in 24 glioma tissue samples and matched adjacent normal tissues, with detailed clinical characteristics summarized in Supplementary Table 5. The results revealed that AL590666.2, POLR2J4, SNHG16, AL359541.1, AC004943.2, and SOX21-AS1 were significantly upregulated in glioma tissues compared to normal tissues (Figure 7A). Based on the prognostic model constructed from the TCGA glioma dataset, we validated the model’s predictive performance using clinical tissue samples from our institution. After calculating the risk scores using the aforementioned formula, patients were classified into high-risk and low-risk groups. Survival analysis demonstrated that the high-risk group had a significantly lower overall survival (OS) compared to the low-risk group (P = 0.020, HR = 3.29 [1.21–8.93]) (Figure 7B). The area under the ROC curve (AUC) for 1-year, 3-year, and 5-year survival rates were 0.713, 0.692, and 0.647, respectively (Figure 7C). Additionally, a time-dependent AUC curve was plotted, indicating that the prognostic model demonstrated good predictive performance for OS in the clinical sample validation cohort (Figure 7D). We performed decision curve analysis (DCA) to evaluate the clinical utility of the LCRlncRNAs prognostic model, which showed that the model has clinical value in predicting survival outcomes (Figure 7E). Finally, the expression levels of AL590666.2, POLR2J4, SNHG16, AL359541.1, AC004943.2, and SOX21-AS1 were significantly elevated in glioma cell lines compared to corresponding normal cell lines (Figure 7F). Collectively, these findings consistently validate the predictive performance of the constructed prognostic model, demonstrating its superior reliability and effectiveness in predicting the prognosis of glioma patients.

### Functional validation of POLR2J4 in glioma progression and chemoresistance

**FIGURE 8:**
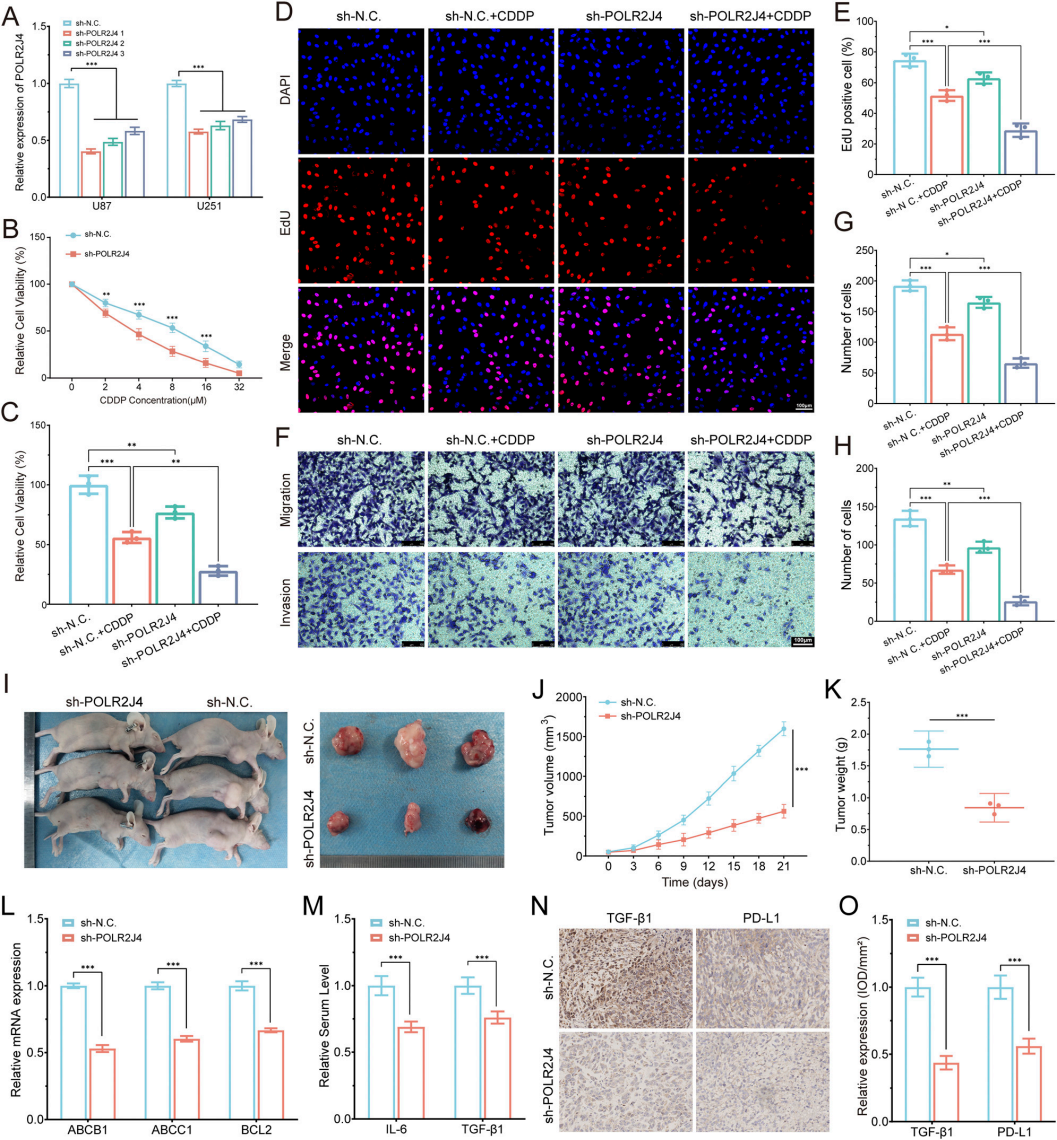
Experimental validation of POLR2J4 function in glioma drug resistance and tumor progression. **(A)** RT-qPCR confirming knockdown efficiency of POLR2J4 in U87 and U251 cells using three shRNAs. **(B)** Dose–response curves showing increased sensitivity to Cisplatin (CDDP) in POLR2J4 knockdown cells. **(C)** Relative cell viability in four groups after treatment with 8 μM CDDP for 48 h. **(D,E)** EdU staining and quantification of proliferative cells across four groups. **(F–H)** Transwell assays evaluating cell migration and invasion, with quantification of migrated and invaded cells. **(I–K)** *In vivo* tumor growth analysis in nude mice (sh-N.C. vs. sh-POLR2J4) using U87 cells, with representative images **(I)**, growth curves (J, n = 3), and final tumor weights **(K)**. **(L)** RT-qPCR analysis of chemoresistance-related genes (ABCB1, ABCC1, BCL2) in U87 cells, demonstrating significant downregulation in the POLR2J4 knockdown group. **(M)** Serum ELISA analysis of pro-tumorigenic cytokines IL-6 and TGF-β1 in tumor-bearing nude mice, indicating reduced circulating levels after POLR2J4 knockdown. **(N,O)** IHC staining and semi-quantitative analysis (IOD/mm^2^) of TGF-β1 and PD-L1 in xenografts, indicating decreased expression in knockdown tumors. *p < 0.05, **p < 0.01, ***p < 0.001.

Among the six prognostic LCRlncRNAs identified by our risk model, POLR2J4 was selected for functional validation due to its strong association with poor prognosis, high expression in glioma tissues and cell lines, and robust correlation with immune checkpoint activation and chemoresistance-related signatures. To explore its biological function, we first confirmed efficient knockdown of POLR2J4 using three independent shRNAs in U87 and U251 cell lines (Figure 8A). Given that Cisplatin (CDDP), a DNA-damaging chemotherapeutic agent, exhibited significantly higher predicted sensitivity in the high-risk group (p < 2.22e−16), we selected it for downstream drug response validation. CCK-8 assays demonstrated that POLR2J4 knockdown significantly enhanced CDDP sensitivity, as evidenced by a steeper reduction in cell viability across a concentration gradient (Figure 8B). When treated with 8 μM CDDP for 48 h, POLR2J4-silenced cells showed a markedly lower viability than either treatment alone, suggesting a synergistic effect (Figure 8C). EdU incorporation assays revealed that POLR2J4 knockdown significantly impaired glioma cell proliferation, which was further exacerbated by CDDP exposure (Figures 8D,E). Transwell assays confirmed that POLR2J4 silencing suppressed cell migration and invasion, with an enhanced inhibitory effect observed in the combination group (Figures 8F–H). *In vivo*, subcutaneous xenograft models using U87 cells demonstrated that tumors derived from POLR2J4-knockdown cells exhibited significantly reduced volumes and final weights compared to controls (Figures 8I–K). Tumor growth curves further confirmed sustained growth suppression in the sh-POLR2J4 group. Collectively, these findings establish POLR2J4 as a tumor-promoting lncRNA in glioma that enhances cell proliferation, invasion, and resistance to CDDP. Its silencing inhibits malignant phenotypes both *in vitro* and *in vivo*, highlighting its potential as a therapeutic target.

### Mechanistic validation of POLR2J4-mediated drug resistance and immunosuppressive microenvironment

To elucidate the underlying mechanisms of POLR2J4-mediated chemoresistance, we performed RT-qPCR analysis of key drug resistance genes. POLR2J4 knockdown significantly reduced the mRNA expression of ABCB1, ABCC1, and BCL2 in U87 cells (Figure 8L). *In vivo*, serum ELISA analysis of pro-tumorigenic cytokines revealed that POLR2J4 knockdown tumors exhibited significantly decreased levels of IL-6 and TGF-β1 (Figure 8M). Immunohistochemical staining of tumor sections demonstrated that POLR2J4 knockdown markedly reduced the expression of TGF-β1 and PD-L1 compared to controls (Figure 8N). Semi-quantitative analysis of IHC staining confirmed significantly decreased IOD/mm^2^ values for both TGF-β1 and PD-L1 in the POLR2J4 knockdown group (Figure 8O). Collectively, these findings establish POLR2J4 as a tumor-promoting lncRNA in glioma that enhances cell proliferation, migration, invasion, and chemoresistance through upregulation of efflux transporters, anti-apoptotic pathways, and immunosuppressive cytokines. Its silencing inhibits malignant phenotypes both *in vitro* and *in vivo* and remodels the immunosuppressive microenvironment, highlighting its potential as a therapeutic target.

## Discussion

Gliomas are highly malignant and invasive brain tumors, with typically poor prognosis for patients. Despite advancements in treatment methods in recent years, their efficacy remains limited. The response of gliomas to immunotherapy is heavily influenced by the tumor immune microenvironment, and biomarkers for predicting immunotherapy outcomes are still unclear. With the growing understanding of immune microenvironments and tumor biological mechanisms, lysine crotonylation, a novel histone modification, has attracted considerable attention (Zhang et al., 2023). Crotonylation, by regulating gene expression, particularly immune-related genes, may play a crucial role in tumor immune evasion and immune microenvironment remodeling (Besermenji and Petracca, 2024). LCRlncRNA, a downstream effector of crotonylation, has been increasingly recognized for its close association with tumorigenesis, progression, and modulation of the immune microenvironment (Yang B. et al., 2024). Although the potential clinical application of LCRlncRNA has been observed in various tumors, its specific role in gliomas remains underexplored. Therefore, this study aims to construct a prognostic model based on LCRlncRNA and further investigate its role in the glioma immune microenvironment and its predictive value for immunotherapy responses.

In this study, we first performed expression analysis of LCRlncRNAs in glioma samples using the TCGA database, and constructed a risk score model based on LCRlncRNAs using univariate and multivariate Cox regression analysis. Through LASSO regression (with λ value determined by 10-fold cross-validation and minimum error at 0.023), we identified six LCRlncRNAs (AL590666.2, POLR2J4, SNHG16, AL359541.1, AC004943.2, SOX21-AS1) associated with prognosis. These lncRNAs may play significant roles in glioma development and progression, consistent with existing literature. For instance, high expression of POLR2J4 has been reported to be associated with poor prognosis in various malignancies (Lu et al., 2019; Wu et al., 2023), while SNHG16 promotes glioma tumorigenesis via activation of the PI3K/AKT pathway and the miR-373/EGFR axis (Zhou et al., 2020). LncRNA AC004943.2 may contribute to tumor progression through regulation of the miR-135a-5p and PTK2/PI3K axis (Zhu et al., 2024), while SOX21-AS1 enhances glioma cell proliferation and invasion by sponging miR-144-3p to upregulate PAK7 expression (Gai and Yuan, 2020). Our study further validates the value of these lncRNAs as potential prognostic biomarkers and suggests that they may influence tumor biology by modulating key signaling pathways, such as PI3K-Akt.

To further verify the functional role of LCRlncRNAs, we focused on POLR2J4, the most representative high-risk lncRNA in our model. Functional assays revealed that POLR2J4 knockdown in glioma cell lines significantly reduced cell proliferation and DNA synthesis, as evidenced by EdU assays, and suppressed both migration and invasion capabilities, as shown by Transwell assays. Importantly, silencing POLR2J4 markedly increased cellular sensitivity to cisplatin, a DNA-damaging chemotherapeutic agent predicted by our model to be more effective in high-risk patients. This was reflected by steeper reductions in viability in both gradient and fixed-dose CCK-8 assays. *In vivo*, xenograft models demonstrated that tumors derived from POLR2J4-silenced cells exhibited significantly decreased growth and weight, confirming its tumor-promoting effect in glioma. These findings strongly support the oncogenic role of POLR2J4 and highlight its potential as both a prognostic biomarker and a therapeutic target involved in proliferation and chemoresistance regulation. Mechanistically, this may be linked to the activation of the PI3K-Akt and DNA repair pathways, though further mechanistic validation is warranted.

Using these lncRNAs, we established a risk score model. To validate the model’s clinical feasibility, we performed external validation on 24 clinical glioma samples, showing that patients in the high-risk group had significantly lower overall survival compared to those in the low-risk group (p = 0.020). Moreover, the risk score model demonstrated high predictive accuracy for 1-year, 3-year, and 5-year survival (AUC >0.8), and the prognostic nomogram (C-index = 0.892) further enhanced its clinical utility. This finding is consistent with previous studies on prognostic models based on other molecular markers, such as m6A-related lncRNAs (Xu et al., 2024; Qin et al., 2023a), indicating the universal applicability of multi-omics models in glioma prognosis stratification.

Functional enrichment analysis revealed potential mechanisms underlying LCRlncRNAs. Differentially expressed genes (DEGs) in the high-risk group were significantly enriched in biological processes related to leukocyte-mediated immune response, extracellular matrix organization, and T-cell activation regulation. KEGG pathway analysis highlighted the activation of the cell cycle, PI3K-Akt, and TNF signaling pathways. The cell cycle and PI3K-Akt pathways are particularly associated with tumor cell proliferation, migration, and survival (Ren et al., 2022; Mohamed et al., 2022), while NF-kappa B and TNF signaling pathways are linked to immune responses and inflammation (Zhao et al., 2022; Lei et al., 2020; Qin et al., 2024a). Thus, the activation of these signaling pathways may drive malignant progression in glioma and promote immune evasion. Notably, although the immune score was higher in the high-risk group, the prognosis was poorer, possibly due to the accumulation of immunosuppressive cells (e.g., Tregs, M2 macrophages) (Zhang and Zhang, 2020; Qin et al., 2023b; Han et al., 2023). For example, M2 macrophages suppress antitumor immune responses by secreting IL-10 and TGF-β (Han et al., 2023), while Tregs facilitate immune evasion by inhibiting effector T-cell function (Qin et al., 2023b). This paradox suggests that the level of immune cell infiltration alone cannot reflect the functional status of the immune microenvironment and necessitates further analysis of immune cell subtypes. Additionally, the high expression of immune checkpoint genes (CD274, CTLA4, LAG3) in the high-risk group further supports the immunosuppressive phenotype, potentially impairing immunotherapy responses via pathways like PD-1/PD-L1 (Zhang and Zhang, 2020).

The PD-L1/PD-1 axis (CD274/PDCD1) and CTLA4 are currently considered core targets in glioma immunotherapy. Clinical studies have demonstrated that PD-L1–positive patients benefit significantly from combination therapy with PD-1 inhibitors (such as pembrolizumab) and CTLA4 inhibitors (such as ipilimumab), which markedly improves survival in recurrent glioblastoma (Duerinck et al., 2024). LAG3 and HAVCR2 (TIM-3) have emerged as important targets in the context of immunotherapy resistance in glioma (Mair et al., 2021; Ding et al., 2022). Preclinical studies have shown that dual blockade of LAG3 and PD-1 can significantly suppress glioma progression (Liu et al., 2020). Recent research indicates that SIGLEC15 is specifically overexpressed in the immunosuppressive microenvironment of glioma and is associated with the polarization of tumor-associated macrophages (TAMs), making it a promising pan-cancer immunotherapeutic target (Wang J. et al., 2023). Although ITPRIPL1 has been less studied in glioma, it has been reported in breast and lung cancers to influence therapeutic response by regulating immune checkpoint molecules, synergizing with PD-L1 to suppress T cell function, and recruiting M2 macrophages (Deng et al., 2023; Krasnyi et al., 2023). This suggests ITPRIPL1 as a novel target for overcoming immunotherapy resistance in glioma, for example, through the development of ITPRIPL1/PD-L1 bispecific antibodies. PDCD1LG2 (PD-L2) mediates immune suppression through TAMs and forms a compensatory resistance axis with PD-L1 (Sumitomo et al., 2022; Umezu et al., 2019); its expression is significantly associated with poor patient survival and immune checkpoint blockade (ICB) resistance. Therefore, targeting PD-L2 may help overcome the therapeutic limitations of PD-1/PD-L1 monotherapy and provide a new avenue for combination immunotherapy.

Regarding the prediction of immunotherapy response, this study is the first to combine LCRlncRNA risk scores with biomarkers such as TMB and MSI. The results showed that high-risk patients had higher TMB and MSI scores, but their prognosis was worse. This phenomenon may be related to the mechanism of “high mutational burden associated with immune escape through immune editing” (Qin et al., 2024b; Qin et al., 2024c), in which tumors evade T-cell recognition by accumulating mutations. Moreover, the positive correlation between LCRlncRNA risk score and HLA and MMR genes (r = 0.62, p < 0.001) suggests that LCRlncRNA may influence immune surveillance by modulating antigen presentation and DNA repair pathways (Aptsiauri and Garrido, 2022; Fu et al., 2023). Taken together, these results highlight the intricate and sometimes paradoxical relationship between tumor immunogenicity and immune evasion, underscoring the complexity of the immune microenvironment in gliomas. This complexity emphasizes the need to integrate multi-omics features for precise risk stratification. Indeed, these findings are consistent with previous studies that constructed lncRNA-based prognostic signatures in various cancers, such as hepatocellular carcinoma and triple-negative breast cancer (He et al., 2025; Ye et al., 2023). Moreover, the PI3K-Akt signaling axis and sphingolipid metabolism, which are enriched in our high-risk group, have been implicated in both cardiotoxicity alleviation and immunotherapeutic response modulation in GBM (Wang L. et al., 2023; Wang et al., 2024). Consistent with our observation of an immunosuppressive microenvironment in high-risk gliomas, recent work has further underscored the interplay between immune checkpoint expression and TME remodeling in GBM (Huang et al., 2023). Together, these studies support the translational potential of integrating lncRNA-based models and immunogenomic profiling to inform precision therapy strategies in glioma and other cancers.

Despite the integrative multi-omics analysis and functional validation, this study has several limitations. First, the glioma samples used for model construction were derived from the TCGA cohort, which predominantly represents Western populations; thus, external validation in multi-center, ethnically diverse cohorts is needed. Second, although immune cell infiltration was assessed using three established algorithms (CIBERSORT, ssGSEA, ESTIMATE), direct experimental validation in terms of comprehensive immune cell profiling was not performed. However, we did perform IHC analysis of key immunosuppressive markers (PD-L1, TGF-β1) in xenograft tissues, as well as serum ELISA analysis of IL-6 and TGF-β1, which partially supports the predicted immunosuppressive microenvironment. Future studies should incorporate immunocompetent or humanized models and perform flow cytometry or immunofluorescence to fully validate immune cell infiltration patterns. Third, while our EdU, Transwell, CCK-8, and xenograft assays confirmed that POLR2J4 promotes glioma proliferation, migration, and chemoresistance, the molecular mechanisms—such as its regulation of PI3K–Akt signaling or immune checkpoints—remain to be elucidated. Finally, although high-risk patients appeared more responsive to cisplatin and other agents, the underlying mechanisms, including potential links to DNA repair or MGMT methylation, require further investigation.

## Conclusion

This study integrates multi-omics analysis with clinical sample validation and functional experiments, revealing the critical role of lysine crotonylation–related lncRNAs (LCRlncRNAs) in glioma prognosis and immune regulation. Six prognostic LCRlncRNAs (AL590666.2, POLR2J4, SNHG16, AL359541.1, AC004943.2, and SOX21-AS1) were identified and used to construct a robust risk model, which stratified patients into distinct prognostic groups and was independently validated in a clinical cohort (1-/3-/5-year AUC >0.7; HR = 3.29, p = 0.020). High-risk patients exhibited immunosuppressive features, including increased Tregs, M2 macrophage infiltration, and elevated PD-L1 expression, suggesting these lncRNAs contribute to therapeutic resistance via PI3K–Akt signaling and immune checkpoint regulation. Functional assays confirmed that POLR2J4 promotes glioma progression and cisplatin resistance, while mechanistic studies showed that POLR2J4 knockdown downregulates key drug resistance genes (ABCB1, ABCC1, BCL2), reduces serum levels of IL-6 and TGF-β1, and suppresses TGF-β1 and PD-L1 expression in tumor tissues, underscoring its role in shaping an immunosuppressive, drug-resistant microenvironment. Although further mechanistic exploration and multi-center validation are warranted, our findings provide a solid foundation for LCRlncRNA-based precision prognostication and targeted therapy in glioma.

## Data Availability

The original contributions presented in the study are included in the article/Supplementary Material, further inquiries can be directed to the corresponding authors.
