# Supplementary material for: Characterization of lysine crotonylation-related lncRNAs for prognostic assessment and immune response in glioma
Source: Front Pharmacol. 2025 Jun 30;16:1573694. doi: 10.3389/fphar.2025.1573694 (PMC12256513; doi:10.3389/fphar.2025.1573694)
Supplement: Supplementary file 1 [file DataSheet1.zip › Supplementary Figures.docx]

Supplementary Figures


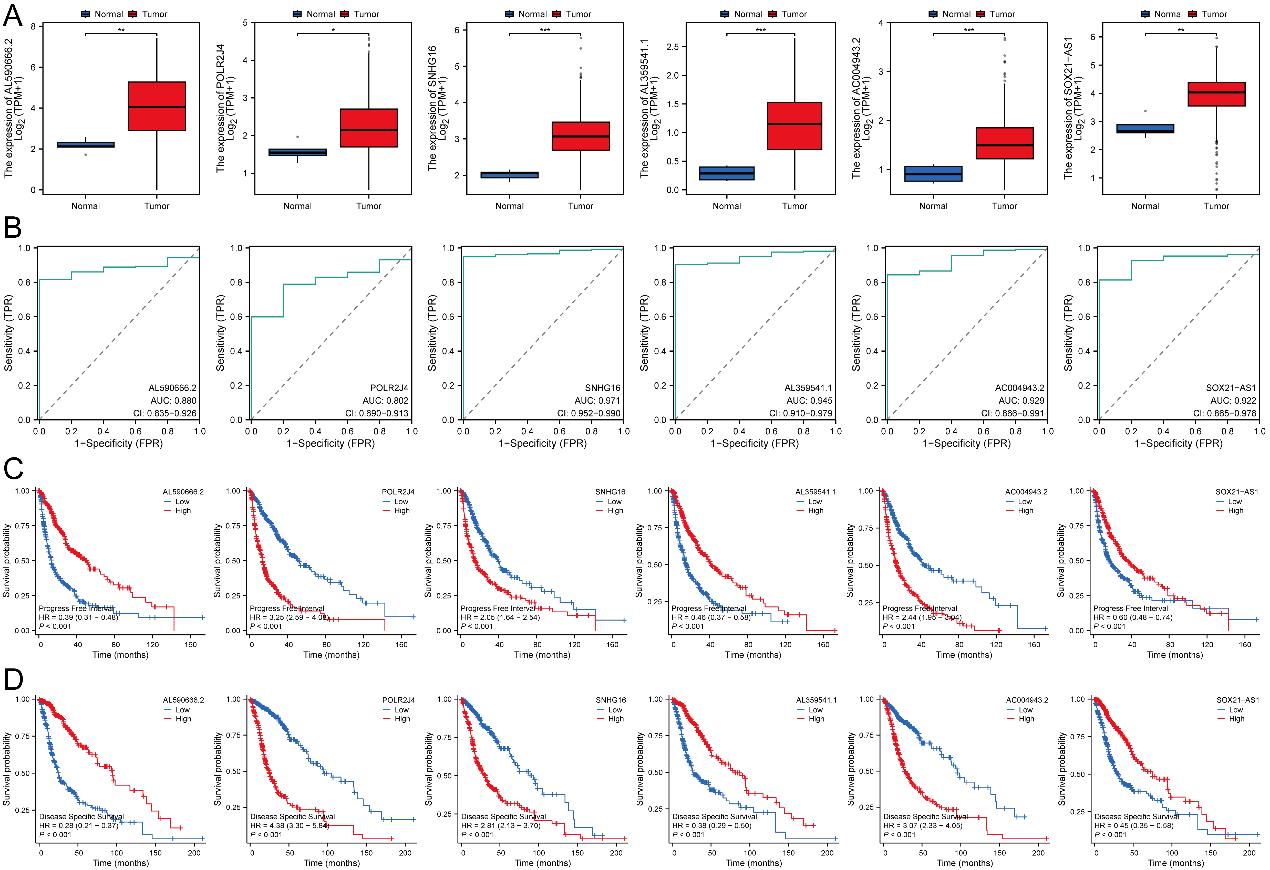


**Supplementary Figure 1.** Expression and Prognostic Value of Prognostic-Related LCRlncRNAs in Glioma Patients. (A) Comparison of prognostic LCRlncRNAs expression in glioma and normal tissues in the TCGA database. *P < 0.05; **P < 0.01; ***P < 0.001. (B) ROC curves evaluating the diagnostic ability of LCRlncRNAs expression for glioma. (C-D) Survival curves for PFI and DSS comparing high and low expression of prognostic LCRlncRNAs in glioma.


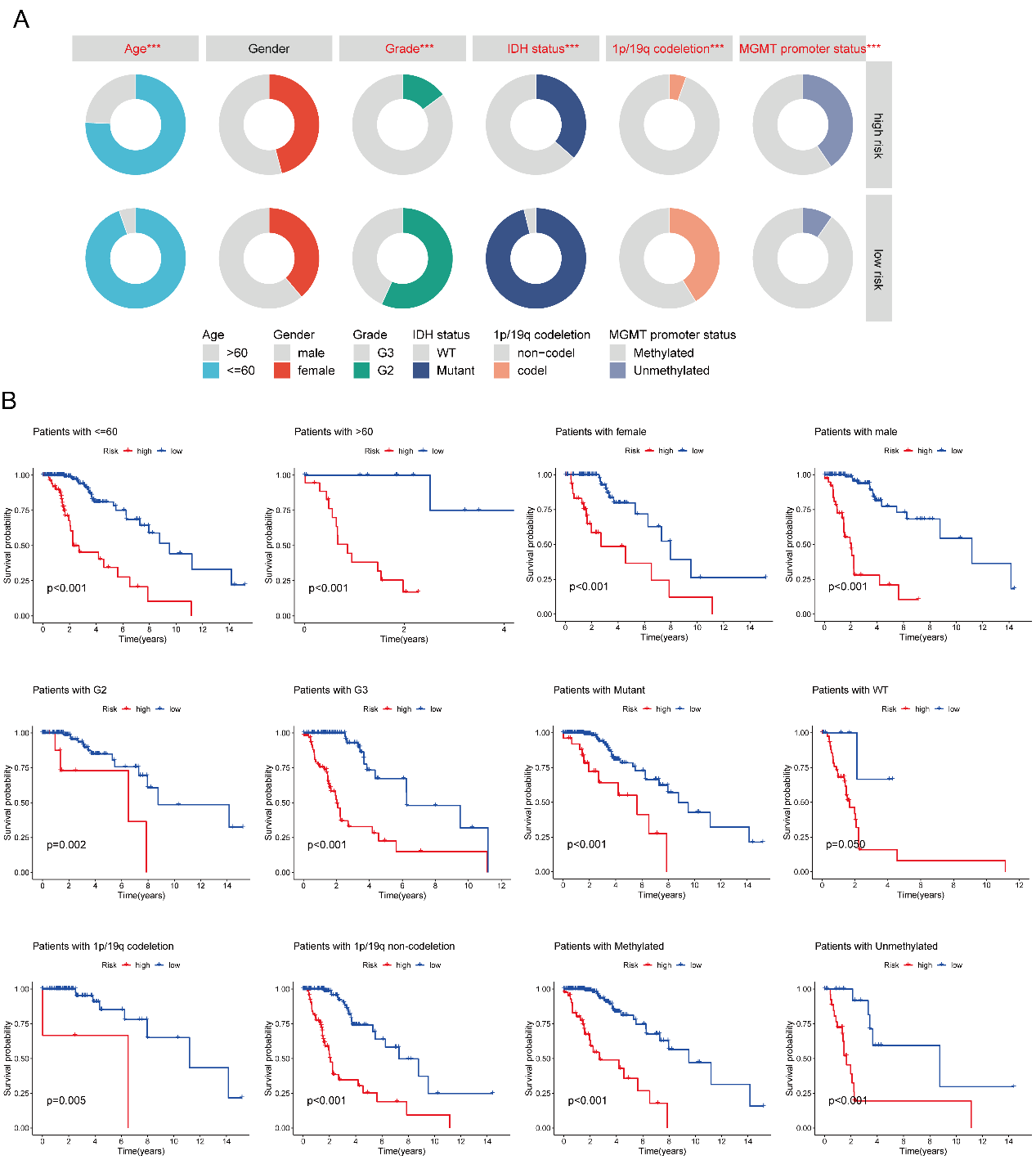


**Supplementary Figure 2.** Association Between Risk Score and Clinicopathological Characteristics. (A) Circos plot showing clinical factors between high and low-risk score groups. (B) Survival curve for high and low-risk score groups in different glioma subgroups.


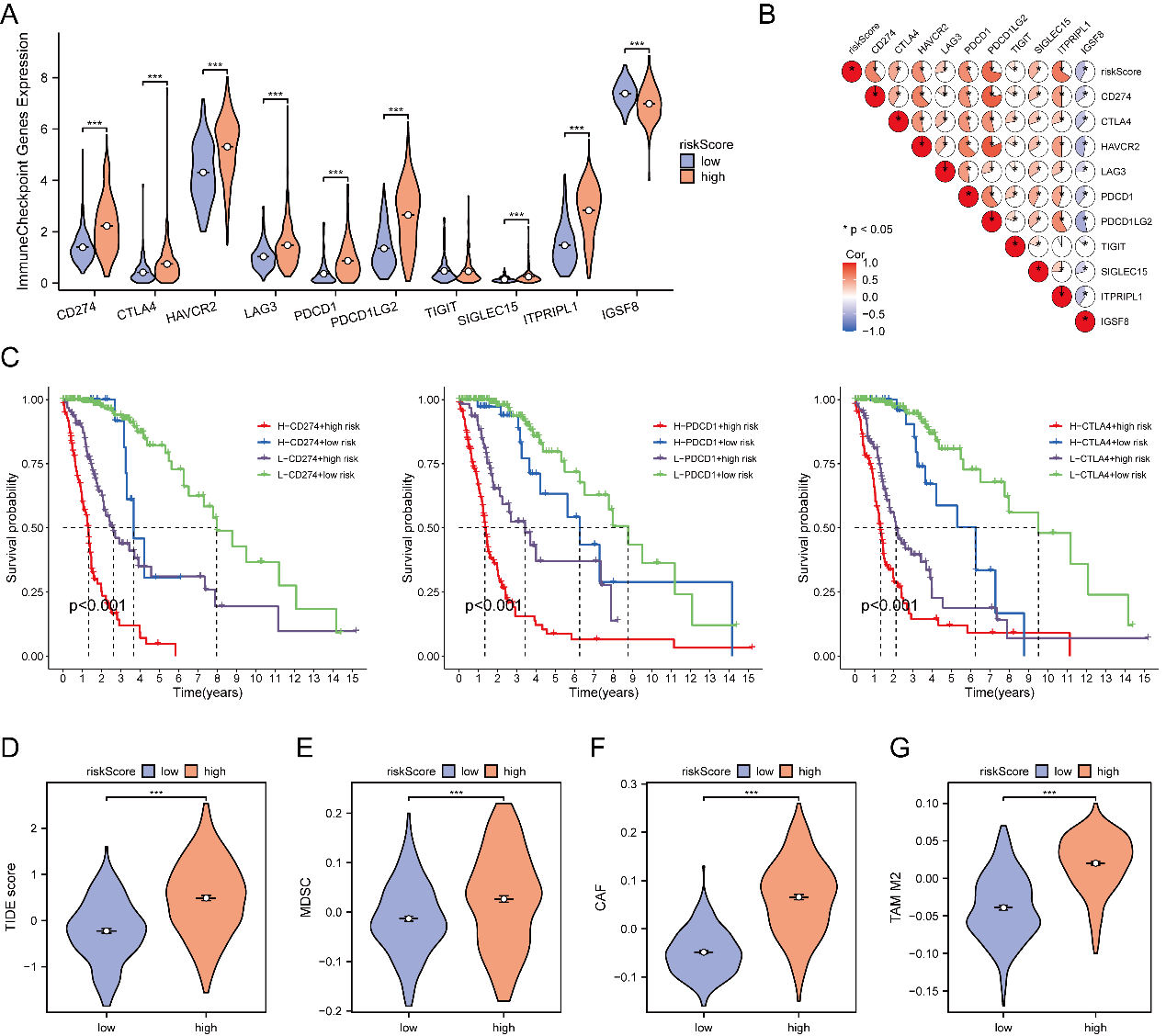


**Supplementary Figure 3.** Immune Therapy Response Analysis. (A) Expression differences of eight immune checkpoint-related genes between high- and low-risk score groups in glioma. (B) Correlation between risk score and immune checkpoint-related genes in glioma. (C) Kaplan-Meier survival curves for four groups classified by risk score and CD274, PDCD1, CTLA. (D-G) TIDE analysis between high- and low-risk score groups in glioma.


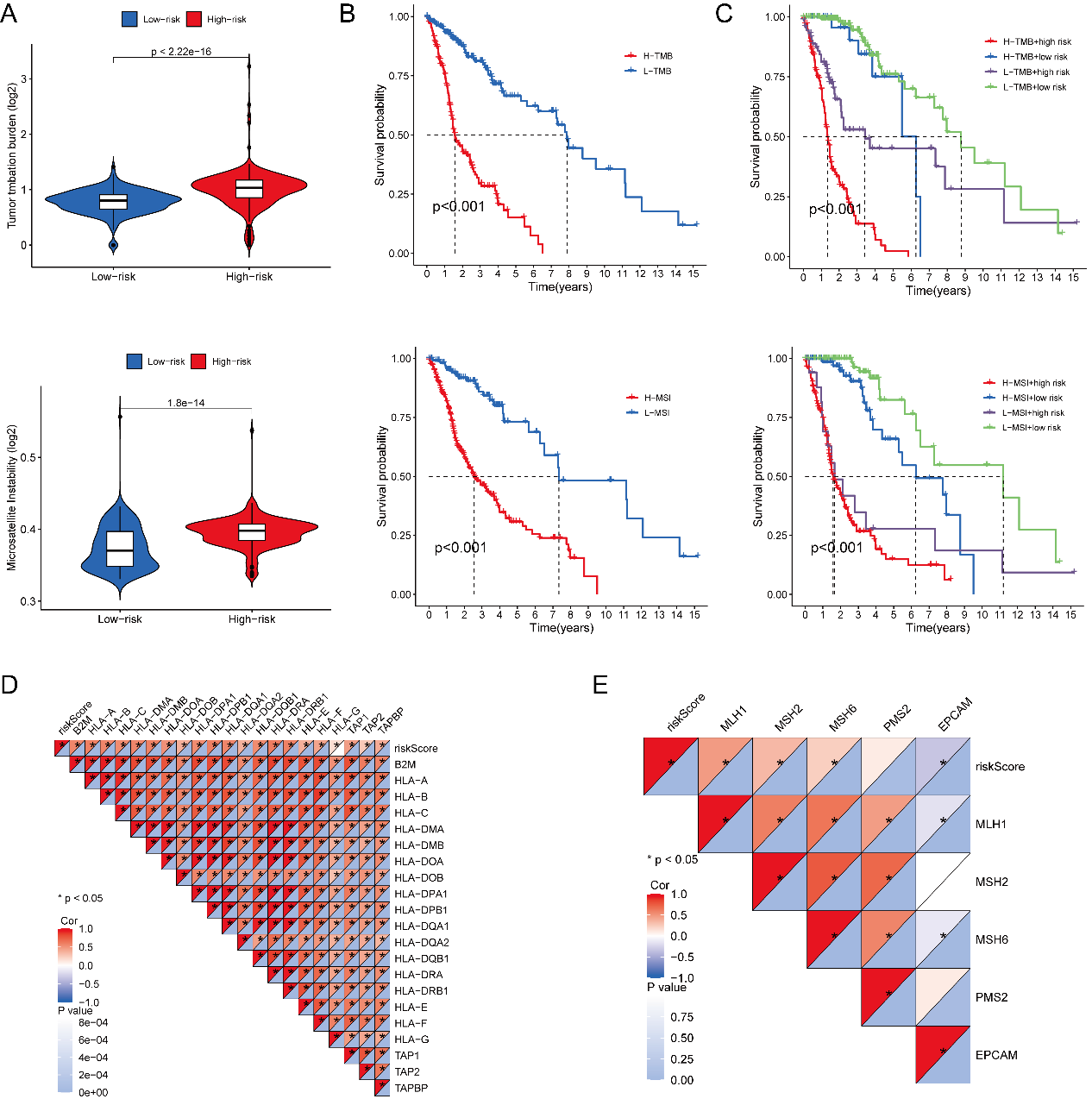


**Supplementary Figure 4.** Predictive Biomarker Analysis for Immune Therapy. (A) Differences in TMB and MSI scores between high and low expression groups. (B) Prognostic analysis of TMB and MSI scores between high and low expression groups. (C) Kaplan-Meier curves for four groups classified by risk score and TMB, MSI in glioma. (D) Association between risk score and HLA. (E) Association between risk score and MMR genes.


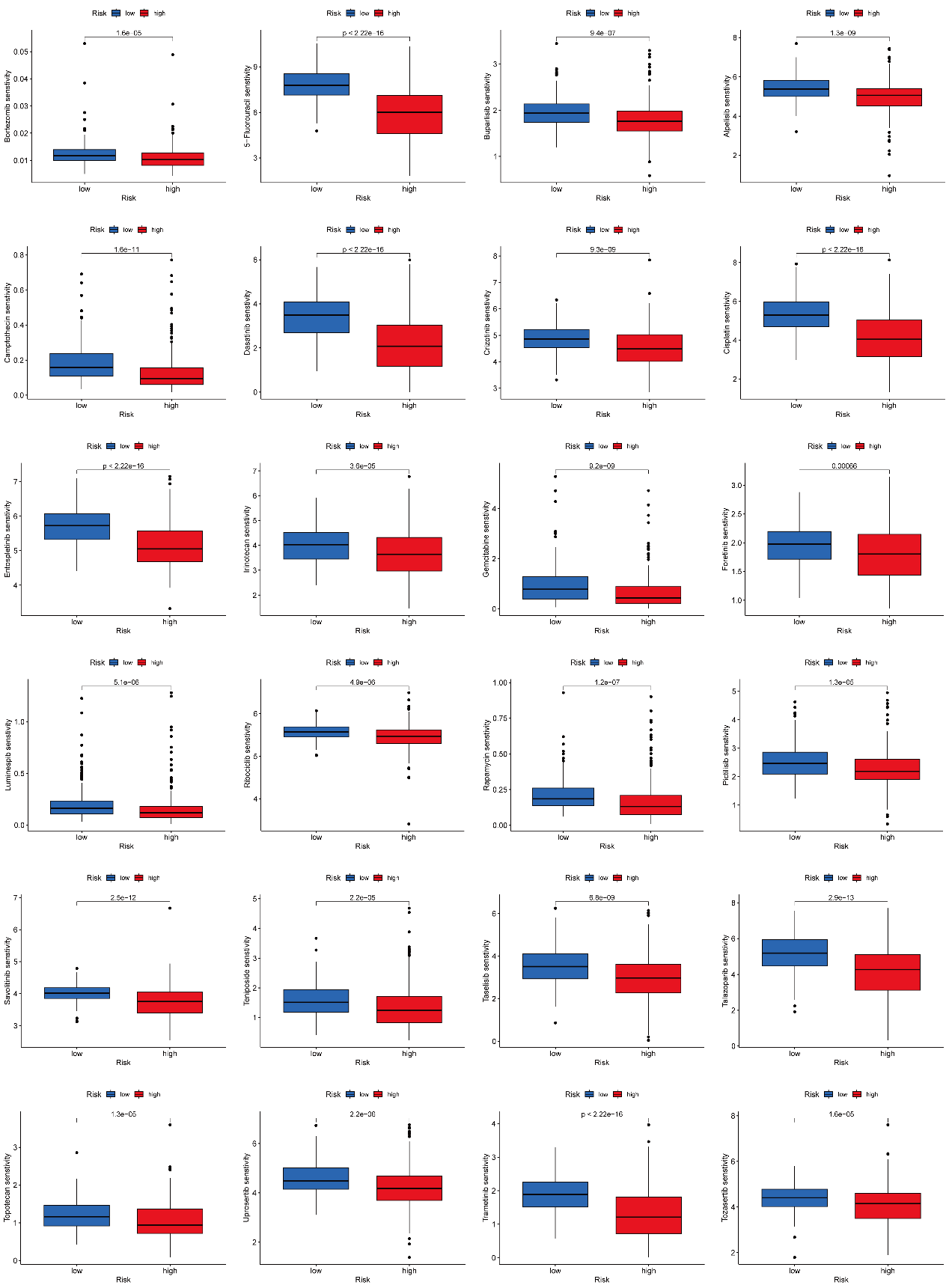


**Supplementary Figure 5.** Chemotherapy Sensitivity Analysis.
