# Supplementary material for: Characterization of lysine crotonylation-related lncRNAs for prognostic assessment and immune response in glioma
Source: Front Pharmacol. 2025 Jun 30;16:1573694. doi: 10.3389/fphar.2025.1573694 (PMC12256513; doi:10.3389/fphar.2025.1573694)
Supplement: Supplementary file 1 [file DataSheet1.zip › Supplementary Table 4.docx]

| Characteristics | high | low | P value |
| --- | --- | --- | --- |
| n | 74 | 206 |  |
| Age, n (%) |  |  | < 0.001 |
| >60 | 18 (6.4%) | 11 (3.9%) |  |
| <=60 | 56 (20%) | 195 (69.6%) |  |
| Gender, n (%) |  |  | 0.286 |
| male | 40 (14.3%) | 126 (45%) |  |
| female | 34 (12.1%) | 80 (28.6%) |  |
| Grade, n (%) |  |  | < 0.001 |
| G3 | 63 (22.5%) | 89 (31.8%) |  |
| G2 | 11 (3.9%) | 117 (41.8%) |  |
| IDH status, n (%) |  |  | < 0.001 |
| WT | 47 (16.8%) | 8 (2.9%) |  |
| Mutant | 27 (9.6%) | 198 (70.7%) |  |
| 1p/19q codeletion, n (%) |  |  | < 0.001 |
| non-codel | 70 (25%) | 121 (43.2%) |  |
| codel | 4 (1.4%) | 85 (30.4%) |  |
| MGMT promoter status, n (%) |  |  | < 0.001 |
| Methylated | 44 (15.7%) | 186 (66.4%) |  |
| Unmethylated | 30 (10.7%) | 20 (7.1%) |  |

Supplementary Table 4. The clinicopathological variables between riskscore high and low groups in TCGA-glioma database
